# Acute airway failure secondary to thyroid metastasis from renal carcinoma

**DOI:** 10.1186/1477-7819-6-14

**Published:** 2008-02-05

**Authors:** Mario Testini, Germana Lissidini, Angela Gurrado, Gaetano Lastilla, Amato Stabile Ianora, Raffaele Fiorella

**Affiliations:** 1 Department of Applications in Surgery of Innovative Technologies; University Medical School of Bari, Italy; 2 Department of Pathology; University Medical School of Bari, Italy; 3 Department of Radiology; University Medical School of Bari, Italy; 4 Department of Otorhinolaryngology; University Medical School of Bari, Italy

## Abstract

**Background:**

Secondary involvement of the thyroid gland by malignant metastases is uncommon. Acute respiratory crisis due to infiltration of the upper airways is a recognised complication of anaplastic thyroid carcinoma or thyroid lymphoma. Renal cell carcinoma is a tumour that metastasizes diffusely and in an unpredictable manner.

**Case presentation:**

We report a case of a 73-year-old man with a painful neck mass, dyspnoea, stridor and dysphonia that was evaluated in emergency. A right radical nephrectomy for renal cell carcinoma was performed 8 years previously. An emergency endotracheal intubation was followed by total thyroidectomy. Histological examination confirmed the diagnosis of thyroid metastasis from renal cell carcinoma.

**Conclusion:**

A literature review regarding emergency treatment for acute respiratory compromise resulting from secondary thyroid tumours was undertaken. Only two cases of metastatic colon cancer and one case of metastatic meningioma requiring emergency thyroidectomy for acute respiratory failure are reported in the literature. This appears to be the first case of emergency surgery performed for acute respiratory compromise due to thyroid metastasis from renal cell carcinoma.

## Background

Acute respiratory obstruction is an uncommon complication of thyroid disease. Most commonly it is due to hemorrhage within a multinodular goiter, bulky mediastinal goiter, anaplastic carcinoma or lymphoma [1-7]. Symptomatic metastases to the thyroid gland are rare, and patients usually complain of a palpable nodule, hoarseness, dysphagia and pain [8,9]. More rarely, it may present with breathing difficulty. In the present report, we describe a patient with thyroid metastases from renal cell carcinoma who presented clinically with acute respiratory failure. Two other similar cases reported in the medical literature are reviewed.

## Case presentation

**Figure 1:**
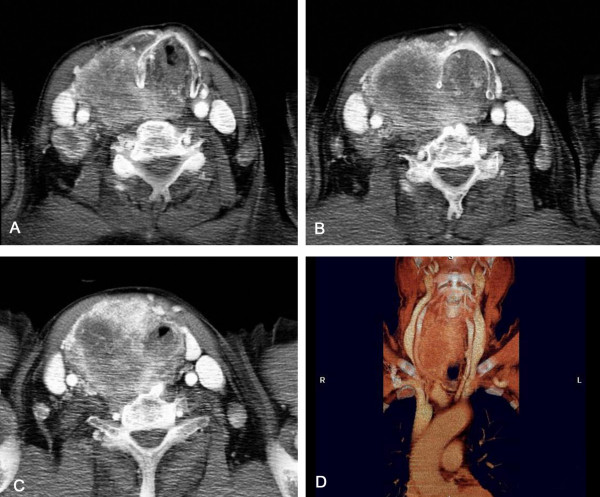
Thyroid metastases due to renal cell carcinoma. Contrast-enhanced computed tomography scan: (A, B, C) axial images and (D) volume-rendered reconstructed image; the right lobe of the thyroid gland shows a non-homogeneous and irregular mass with tracheal involvement. The mass extends into the fatty plane in proximity to the right carotid artery and is also associated with metastatic lymph nodes.

A 73-year-old man was admitted in emergency to the general surgery department with a neck mass, sudden dyspnoea, stridor, dysphonia, and progressively worsening dysphagia. His medical history included a multinodular goiter ans right radical nephrectomy performed 8 years prior due to renal cell carcinoma. At annual follow-up, a CT of the thorax and abdomen was performed and the thyroid mass was also evaluated by ultrasonography and thyroid function tests. Five months earlier, the patient had undergone fine-needle aspiration consistent with multinodular goiter. Three days before admission the patient underwent a total-body CT scan that revealed a thyroid mass with substernal extension involving and obstructing the upper airways, right vocal cord and jugular vein and showed carotid artery compression and displacement, in addition to diffuse lymphadenopathy (Figure 1).

**Figure 2:**
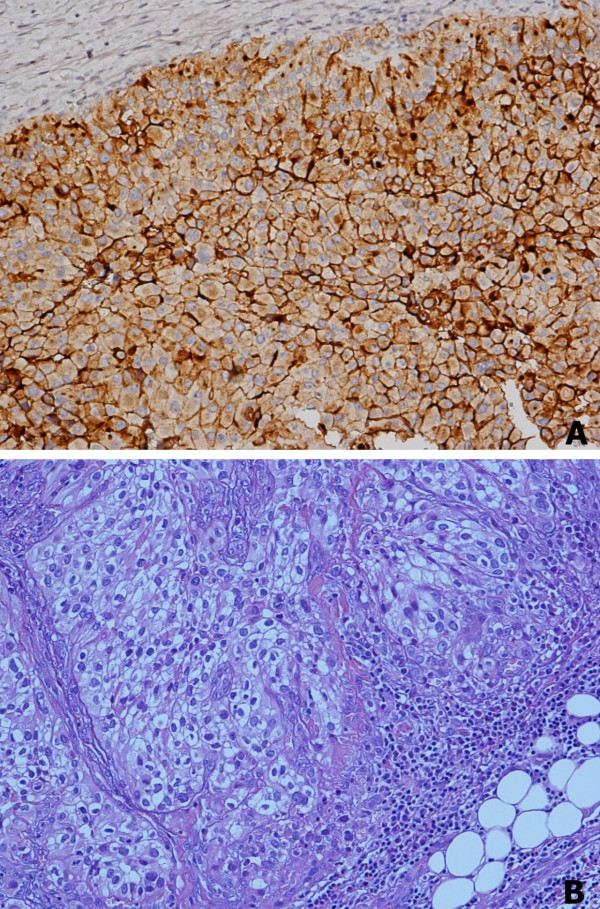
Histological findings. A) Neoplastic cells strongly expressed CD10 antigen (Immunoperoxidase, ×200). B) Histology revealed a diffuse growth of neoplastic cells with an evident clear cytoplasm (hematoxylin and eosin, ×200).

Physical examination revealed a large, painful, diffuse, and predominantly right-sided thyroid tumour. Thyroid function tests were normal. A flexible laryngoscopy revealed right vocal cord palsy and left vocal cord paresis, with a nearly total reduction of the laryngeal lumen. Emergency endotracheal intubation was performed, followed by total thyroidectomy using loupe magnification [10] with lymph node dissection. The surgery was completed by a tracheotomy, given the evident tracheomalacia. The thyroid gland was found to have been fully replaced by a soft yellow mass weighing 40 g and 8.5 × 5.5 × 4.5 cm large, with indistinct borders infiltrating peri-thyroid muscles and involving three lymph nodes. Histological examination revealed a carcinoma composed mainly of clear cells with scanty oxyphil cells. Neoplastic cells showed large pleomorphic nuclei and frequent mitoses. Lymphatic and vascular invasions were common findings. Immunohistochemistry revealed strong and diffuse expression of CD10 antigen (Figure 2A–B) was positive for Vimentin and negative for thyroid transcription factor-1 staining. Histology and immunohistochemistry were characteristic of metastatic clear renal cell carcinoma.

The patient had an uneventful postoperative course and was discharged after 10 days. Despite palliative chemotherapy, the disease progressed and the patient died 7 months later.

## Discussion

The diagnosis is often incidental, resulting from histological examination of single nodule or multinodular goitre. Although our case produced unsuccesful results, fine-needle aspiration cytology plays an important role in diagnosing thyroid metastasis and is recommended by some authors. Secondary malignancies of the gland are believed to comprise less than 1% of thyroid cancers [8]. The overall incidence of metastases to the thyroid varies from 1.2% in unselected autopsy series to 24% in autopsy of patients with widespread malignant neoplasms [11].

Autopsy series reveal that thyroid metastases are most commonly due to breast, lung, melanoma, renal, and gastrointestinal carcinomas [8,11]. However, when only clinically relevant metastases are considered, the incidence of renal cell carcinoma increases to 50% [8].

The thyroid gland is highly vascularized and its rich vascular supply inibits the embolization of tumoural cells. The reduced arterial supply and tissue iodine concentration of adenomatous gland, as in this case report, have been previously recognised as risk factors for the growth of metastatic malignant cells [8,9,11]. Renal cell carcinoma can metastasize to the thyroid bypassing the lungs via the valveless paravertebral venous plexus of Batson [12], exceptionally representing the first manifestation of widespread tumour dissemination. Recurrence may develop several years after the original diagnosis of the primary lesion, without specific signs or symptoms. Moreover, no sensitive tests assist in the preoperative diagnosis, as was demonstrated in this report by a standard fine-needle aspiration biopsy [11,13,14] and absence of thyrotoxicosis that is contrary to previous reports [15].

Acute respiratory crisis caused by infiltration of the upper airways is a recognised complication in both the anaplastic thyroid carcinoma and in local squamous cell malignancies [16]. To investigate cases similar to ours, we conducted a Medline search from 1966 to 2007 using the key words "renal cell carcinoma with thyroid/acute airway failure/emergency surgery, and thyroid metastases with acute airway failure/emergency surgery/emergency treatment" in the title and abstract fields. Results showed that emergency surgery for acute respiratory failure due to secondary thyroid tumours was needed only in two cases of metastatic colon cancer [17] and in one case of metastatic meningioma [18]. The present report illustrates an additional case of acute airway obstruction resulting from thyroid metastatic disease. This case expands the spectrum of clinical manifestations described for thyroid metastases from renal cell carcinoma.

## Conclusion

Increasing attention to concomitant thyroid disease is mandatory in patients who have undergone nephrectomy for renal cell carcinoma to improve follow-up accuracy and to avoid the rare but dramatic complication described herein. Studies focusing on prophylactic total thyroidectomy in the presence of a diagnosis of multinodular goiter during follow-up of patients with a history of renal cell carcinoma, should be encouraged.

## Competing interests

The author(s) declare that they have no competing interests.

## Authors' contributions

**MT: **the surgeon; approved the final version of the manuscript for publication. **GL **responsible for critical revision of scientific content **AG **drafted the manuscript. **GL **performed histopathological and immunohistochemical analyses and contributed to the pathology content. **ASI **performed the CT examination. **RF **contributed substantially to manuscript conception and design.

All authors read and approved the final version of the manuscript.
